# Meningeal lymphatic dysfunction in Alzheimer’s disease: molecular mechanisms, clinical implications, and future perspectives

**DOI:** 10.1186/s13024-026-00962-0

**Published:** 2026-06-11

**Authors:** Lian Liu, Jiang-Hui Li, Yu-Di Bai, Yan-Jiang Wang, Xian-Le Bu

**Affiliations:** 1 Department of Neurology and Centre for Clinical Neuroscience, Daping Hospital, Third Military Medical University, Chongqing, China; 2 Chongqing Key Laboratory of Ageing and Brain Diseases, Chongqing, China; 3 Key Laboratory of Geriatric Cardiovascular and Cerebrovascular Disease, Third Military Medical University, Chongqing, China; 4 Institute of Brain and Intelligence, Third Military Medical University, Chongqing, China; 5 State Key Laboratory of Trauma and Chemical Poisoning, Third Military Medical University, Chongqing, China

**Keywords:** Meningeal lymphatic vessels, Alzheimer’s disease, Amyloid-beta, Tau, Neurodegeneration, Neuroimmune modulation, Myelin function, Intervention

## Abstract

The meningeal lymphatic system has recently emerged as a critical regulator of brain homeostasis, facilitating cerebrospinal fluid drainage, metabolic waste clearance, and immune cell trafficking. Accumulating evidence now implicates meningeal lymphatic dysfunction as a pivotal contributor to the pathogenesis of Alzheimer’s disease (AD). This review critically evaluates current neuroimaging techniques for assessing meningeal lymphatic function in humans, highlighting their technical limitations in capturing dynamic pathological changes specific to AD. We summarize recent advances demonstrating that meningeal lymphatic impairment exacerbates key AD hallmarks—including amyloid-β (Aβ) and tau deposition, neuroimmune dysregulation, and myelin degradation—collectively accelerating disease progression. Building on these insights, we systematically analyse emerging therapeutic strategies aimed at enhancing meningeal lymphatic function, such as pharmacological approaches (e.g., vascular endothelial growth factor C (VEGF-C)-mediated lymphangiogenesis), physical interventions (e.g., transcranial photobiomodulation), and surgical techniques (e.g., cervical lymphaticovenous anastomosis). However, significant challenges remain, including the scarcity of direct human evidence linking meningeal lymphatic dysfunction to AD and the lack of standardized, noninvasive assessment tools. To address these gaps, we propose future fundamental and clinical research directions for meningeal lymphatic vessels and AD. By bridging mechanistic insights with translational applications, this review highlights the role of the meningeal lymphatic system as a promising yet underexplored target for AD modification.

## Background

Alzheimer’s disease (AD) is a prevalent neurodegenerative disorder and represents the leading cause of dementia among elderly people. Its neuropathological hallmarks include extracellular senile plaques, which are composed of aggregated amyloid-β (Aβ) peptides, and intracellular neurofibrillary tangles, which consist of hyperphosphorylated tau protein. However, the mechanisms driving the aberrant accumulation of Aβ and tau in the AD brain remain elusive, and effective therapeutic interventions are still lacking. Current AD immunotherapies (e.g. lecanemab and donanemab) can delay cognitive decline by approximately 30% in patients with early AD. Furthermore, their clinical utility is limited by a significant incidence of adverse effects [1–3]. Consequently, elucidating the pathogenesis of AD and developing safer, more effective therapeutic strategies remain critical and unmet challenges in the field.

Meningeal lymphatic systems have been associated with various central nervous system (CNS) disorders, offering novel perspectives on the pathogenesis and treatment of neurological diseases. Analogous to peripheral lymphatic drainage, which eliminates macromolecules and metabolic waste, the meningeal lymphatic network serves as a critical pathway for clearing metabolic waste and neurotoxic substances from the brain. This system mediates solute transport, cellular debris, and immune cell trafficking from the cerebrospinal fluid (CSF) and brain parenchyma to the cervical lymph nodes (CLNs) and facilitates clearance of pathological proteins such as Aβ and tau. Moreover, meningeal lymphatic vessels (mLVs) modulate key pathophysiological processes in AD, including intracerebral immune responses, myelin formation, and synaptic plasticity. Given the established association between mLVs function and both AD pathogenesis and progression, this review synthesizes recent advances in the field and proposes novel potential intervention strategies targeting mLVs.

## Function of the meningeal lymphatic system

### Discovery and structure of the mLVs

The presence of mLVs in humans was initially documented by Italian anatomist Paolo Mascagni in 1787, yet this finding has remained largely overlooked for decades [4]. In 2015, research teams led by Jonathan Kipnis and Kari Alitalo independently identified structural entities within the murine dura mater that exhibited molecular phenotypes characteristic of lymphoendothelial cells and were designated mLVs [5, 6]. Building on these foundational murine studies, Daniel Reich’s team visualized functional mLVs in humans and marmosets in 2017 using noninvasive magnetic resonance imaging (MRI) [7]. Later, in 2020, the laboratory of Brant M. Weinstein reported an evolutionarily conserved meningeal lymphatic network in zebrafish, demonstrating functional parallels to the mammalian system [8]. Although conventional lymphatic vessels are absent from the brain parenchyma, the glymphatic system (GS) —a theoretical model proposing that perivascular channels ensheathed by astrocytic endfeet facilitate the efflux of interstitial fluid and solutes from the parenchyma to the mLVs—has been proposed. It should be noted, however, that the glymphatic concept remains a theoretical construct rather than a definitively established anatomical system, and its existence and physiological significance remain subjects of debate. For instance, direct anatomical connections between arterial and venous perivascular spaces have not been conclusively demonstrated, and some investigators have questioned the validity of the underlying evidence [9]. Notwithstanding these conceptual uncertainties, there is general agreement that mLVs not only facilitate drainage of CSF, solutes, and metabolic waste to CLNs but also mediate immune cell trafficking from the CNS to peripheral lymphoid tissues. This dual functionality constitutes a vital mechanism for modulating CNS–peripheral immune crosstalk [5, 6, 10–12]. These findings challenge the historical paradigm of the CNS as an immunologically privileged site devoid of lymphatic drainage. Collectively, these findings underscore the pivotal role of mLVs in brain waste clearance, thereby providing novel insights into the pathogenesis of cerebral metabolic dysfunction in neurodegenerative pathologies such as AD.

**Fig. 1:**
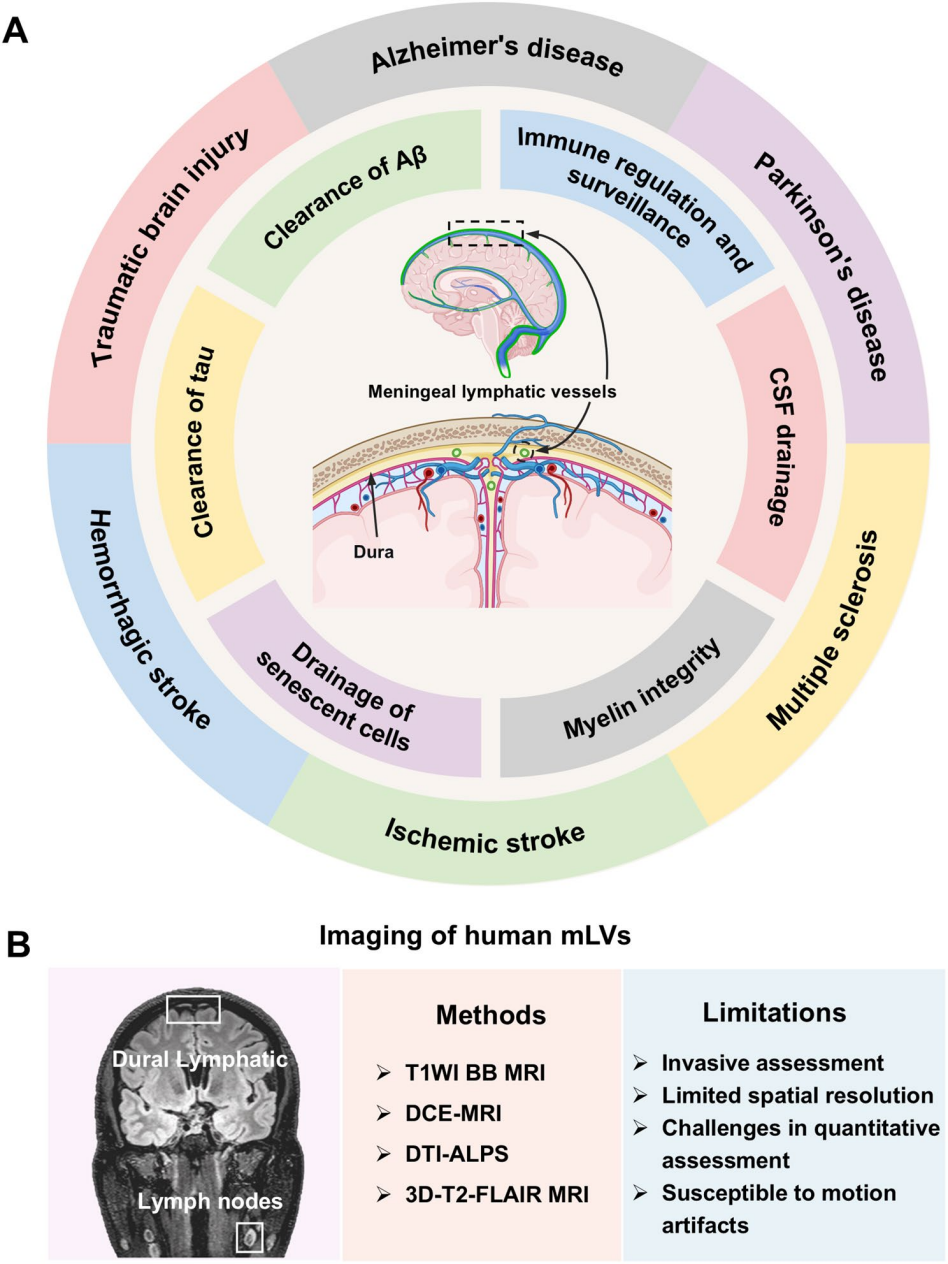
(**A**) The physiological functions of the meningeal lymphatic system and related diseases. (**B**) Current neuroimaging techniques for assessing meningeal lymphatic function and their limitations. MRI adapted from “Non-invasive MR imaging of human brain lymphatic networks with connections to cervical lymph nodes”, by Albayram, M.S. et al., *Nat Commun* 13, 203 (2022), CC BY 4.0. Aβ: β-amyloid; CSF: cerebrospinal fluid; mLVs: meningeal lymphatic vessels; T1WI BB MRI: T1-weighted black-blood MRI; DCE-MRI: dynamic contrast-enhanced MRI; DTI-ALPS: diffusion tensor imaging analysis along the perivascular space; 3D-T2-FLAIR MRI: 3-dimensional T2-weighted fluid-attenuated inversion recovery MRI

Anatomically, the brain is enveloped by three meningeal layers from deep to superficial: the pia mater, arachnoid mater, and dura mater. Investigations into the murine meningeal lymphatic system have revealed that mLVs are primarily situated within the dura mater, exhibiting two key distributions: dorsal mLVs, which run alongside the superior sagittal and transverse sinuses within the dural folds, and basal mLVs, which follow the course of the petrosquamosal and sigmoid sinuses [5, 6, 13] (Fig. 1A). Functionally, the GS first facilitates the clearance of molecules and metabolites from the brain parenchyma to the subarachnoid space via perivascular pathways. These waste products are subsequently drained by mLVs to CLNs, thereby forming a dual-clearance pathway for the removal of CNS waste [14]. Studies have shown that aging induces dorsal mLVs regression, whereas skull-based mLVs display lymphedema-like alterations, including hyperplasia, tortuosity, and dilation [13]. Jacob et al. first mapped the distribution and drainage of mLVs in the anterior cranial fossa (e.g., the cavernous sinus) [15], whereas de Leon et al. identified CSF drainage to CLNs via the superior turbinate region [16]. In rodents, approximately 50% of the CSF is estimated to be drained to CLNs through mLVs [17–19]. Beyond their role in fluid and waste clearance, mLVs also contribute to CNS immune homeostasis by transporting brain-derived proteins and metabolites to CLNs, where these antigens can elicit adaptive immune responses. Further investigation into the structure and distribution of mLVs in the brain is essential, as it provides the foundational knowledge required to understand their functional role in AD.

### Dysregulation of mLVs in aging and neurological disorders

mLVs emerge during the first postnatal week, originate from the foramina of the skull base and vertebral canal, and then progressively extend along the vasculature and cranial/spinal nerves to colonize the CNS-surrounding meninges [20]. During aging, mLVs exhibit progressive structural and functional deterioration. In AD and aged mice, mLVs show reduced coverage, diameter, and branching, along with significantly impaired drainage function [13, 21, 22]. Near-infrared imaging revealed significantly reduced CSF efflux through meningeal lymphatics in aged mice compared with young mice [17]. Human studies corroborate these findings, showing that age-related dysfunction in lymphatic dilation/contractility, which is associated with CLN atrophy and lymphatic vessel thickening, compromises CSF drainage efficiency [23]. Notably, elevated meningeal IFN-γ in aged mice directly induces lymphatic impairment, a phenotype reversible upon IFN-γ antibody neutralization [21]. Furthermore, alterations in genes associated with transmembrane receptor protein tyrosine kinase signalling pathways in lymphatic endothelial cells (LECs) impair their repair and regenerative capacity in aged mice, resulting in the dysfunction of mLVs [13]. Ageing also decreases the contraction frequency and flow velocity in cervical lymphatics, the primary drainage route for mLVs in mice [24]. In AD transgenic mice, attenuated mLVs contractions have been shown to impair meningeal drainage and reduce CSF outflow to CLNs [25]. Using functional photoacoustic microscopy, a ~ 70% reduction in mLVs drainage volume has also been quantified in AD mice [26]. In addition, ablation of mLVs in AD mice exacerbates Aβ deposition in the meninges and brain parenchyma [27]. Conversely, Antila et al. [28] reported that sustained mLVs atrophy or hyperplasia did not significantly alter Aβ deposition or behavioral performance in APP/PS1 and 5xFAD mice, despite affecting CSF flow to cervical lymph nodes, highlighting the complexity and ongoing debate regarding the role of mLVs in Aβ clearance. CLN ligation also aggravates AD-related pathology, neuroinflammation, and cognitive deficits [29]. Dysfunction of mLVs and cervical lymphatics has also been observed in Parkinson’s disease (based on human data [30]), as well as in animal models of stroke [31] and traumatic brain injury [32], underscoring its broad relevance in neurological disorders (Fig. 1A). Although direct clinical evidence in AD patients remains limited, owing to challenges in assessing human meningeal lymphatic function, considerable evidence has demonstrated that mLVs dysfunction is a hallmark of both ageing and neurological disorders. Collectively, these insights establish mLVs as critical mechanistic nodes and promising therapeutic targets for AD and related disorders.

### Imaging strategies for the human meningeal lymphatic system

Human mLVs typically measure 30–100 μm in diameter, although current in vivo imaging techniques are limited by resolution limitations. The principal modalities for the functional evaluation of mLVs include T1-weighted black-blood MRI (T1WI BB MRI), dynamic contrast-enhanced magnetic resonance imaging (DCE-MRI), diffusion tensor imaging analysis along the perivascular space (DTI-ALPS), and two- or three-dimensional fluid-attenuated inversion recovery (FLAIR) MRI (Fig. 1B). A landmark 2017 study first achieved successful mLVs visualization in humans and nonhuman primates through synergistic application of T1WI BB MRI with 2D-FLAIR MRI [7]. Subsequent studies have evaluated mLVs function by assessing the severity of white matter hyperintensity and imaging markers related to cerebral small vessel disease. The results indicate that the imaging manifestations of white matter hyperintensities and vascular lacunes are negatively correlated with the metabolic function of mLVs [33, 34]. Furthermore, quantitative assessments using DCE-MRI revealed a significant reduction in mLVs flow along the superior sagittal and sigmoid sinuses, accompanied by markedly delayed perfusion of deep cervical lymph nodes (dCLNs), in patients with idiopathic PD compared with those with atypical PD[30]. DTI-ALPS has proven particularly valuable for evaluating mLVs function. A reduced DTI-ALPS index has been observed in patients with spontaneous intracerebral hemorrhage (sICH), suggesting potential mLVs dysfunction [35]. Moreover, the DTI-ALPS index has been identified as an independent predictor of poor 90-day functional outcomes in sICH patients [35, 36]. DTI-ALPS has been applied to reveal a reduction in the ALPS index across the preclinical, prodromal, and dementia stages of AD, suggesting that glymphatic impairment may precede significant Aβ deposition and could serve as a predictor of cerebral Aβ accumulation, neurodegeneration, and clinical progression in AD [37]. Researchers have established a noncontrast 3D-FLAIR MRI protocol that provides high-resolution mapping of dorsolateral and ventral mLVs along the dural sinuses as well as their anatomical connections to dCLNs [23]. With high resolution, 7T MRI may offer a novel approach for visualizing meningeal lymphatic vessels [38]. Furthermore, although stereoscopic wide-field photoacoustic microscopy has enabled in vivo tracking of mLVs in mice, its application in humans requires further in-depth investigation [26].

In summary, the current assessment of human meningeal lymphatic function primarily relies on MRI-based techniques, each with significant limitations. T1-weighted black-blood MRI and DCE-MRI are invasive procedures requiring intravenous contrast administration, which carries potential risks including allergic reactions and nephrotoxicity, limiting their repeated use in longitudinal studies. 3D-T2-FLAIR MRI offers noninvasive anatomical visualization of dural lymphatic structures; however, recent evidence suggests that high-signal intensities attributed to lymphatics—particularly in the ventral skull base region—may represent artifacts due to incomplete fluid signal suppression caused by local magnetic field inhomogeneity, raising concerns about the specificity of this technique for mLVs identification [39]. Regarding DTI-ALPS, a more critical appraisal is warranted. As highlighted by Ringstad [40], the validity of DTI-ALPS as a marker of glymphatic-meningeal lymphatic function is questionable, as the index reflects water diffusivity in deep white matter where CSF-ISF exchange plays only a minor role. Furthermore, recent studies have demonstrated that ALPS indices are significantly confounded by white matter microstructural features—including fiber crossings, axonal undulations, and dispersion - rather than specifically reflecting perivascular water motion [41]. Consequently, a decreased ALPS index should be interpreted as exactly that, rather than directly equated with “glymphatic dysfunction” [42]. Given the critical role of visualizing mLVs function in evaluating the progression of various central nervous system disorders, there is an urgent need to develop noninvasive, convenient, and more effective clinical tools. In this context, identifying reliable fluid biomarkers that reflect mLVs activity in blood and CSF represents a promising direction for future research. For instance, plasma tau levels have been shown to correlate with meningeal lymphatic function indices [43]. Furthermore, p-tau181 and Aβ have been detected in human cervical lymph nodes [44, 45], suggesting that levels of AD-related proteins in blood or lymph node aspirates may serve as surrogate markers of mLVs drainage efficiency. Future studies should also explore CSF-to-blood clearance rates of labeled tracers as functional biomarkers. Collectively, searching for fluid biomarkers that reliably reflect mLVs function in blood and CSF is a worthwhile endeavor.

## Roles of the meningeal lymphatic system in AD pathogenesis

### Meningeal lymphatic system and Aβ clearance

Impaired clearance of Aβ is a critical upstream event in the pathogenesis of sporadic AD. In animal models, the GS clears parenchymal Aβ through directional CSF/ISF flow, despite the absence of lymphatic vessels in the brain parenchyma [46–53]. However, how the GS clears downstream brain waste remaines unclear. The recent discovery of mLVs has refined the drainage route of solutes via the GS. Located in the dura mater, mLVs receive metabolic waste from the GS and drain CSF from the subarachnoid space (including venous sinuses) to the dCLNs [5, 6]. Multiple animal studies have demonstrated that impaired meningeal lymphatic drainage exacerbates Aβ deposition in AD mice, suggesting that meningeal lymphatic dysfunction may be a contributing factor to AD pathological progression and cognitive decline [27, 54].

Previous research has confirmed that vascular endothelial growth factor C (VEGF-C) promotes lymphatic development by activating the MAPK/ERK and PI3K/Akt downstream signalling pathways by binding to vascular endothelial growth factor receptor 3 (VEGFR-3) [55]. Intracerebral injection of VEGF-C in aged mice enhances meningeal lymphatic drainage and cognitive function [27]. In vitro studies have shown that recombinant human VEGF-C (rhVEGF-C) significantly promotes tubule formation in human lymphatic endothelial cells. Similarly, the intracisternal injection of rhVEGF-C in APP/PS1 mice promotes meningeal lymphangiogenesis and the clearance of soluble Aβ, leading to improved spatial cognitive function [56]. Furthermore, anti-Aβ antibody monotherapy in aged AD mice has limited Aβ clearance efficiency, whereas combining Aβ immunotherapy with mVEGF-C injection restored meningeal lymphatic function and significantly enhanced Aβ clearance. In younger AD mice, the synergistic effect of combined therapy is more pronounced, suggesting that the “integrity of meningeal lymphatic function” may be a key prerequisite for effective Aβ clearance in AD immunotherapy [54]. Additionally, studies have shown that down syndrome critical region 1 (DSCR1) can improve drainage efficiency by promoting dorsal mLVs growth, thereby alleviating AD-related pathology [57].

As downstream conduits for CSF and ISF drainage into the peripheral lymphatic system, cervical lymphatic vessels and CLNs also play important roles in Aβ clearance [17]. Pappolla et al. first demonstrated the presence of Aβ in the cervical and axillary lymph nodes of AD transgenic mice, with Aβ levels in these nodes increasing with age—a phenomenon not observed in other tissues, indicating that Aβ from the brain can flow to CLNs [58]. Similarly, Aβ has been observed in human CLNs [44, 45]. Surgical ligation of dCLNs to block meningeal lymphatic drainage to dCLNs exacerbated Aβ pathology, neuroinflammation, and cognitive deficits in APP/PS1 mice [29]. Conversely, enhancing cervical lymphatic contraction through pharmacological or physical massage approaches effectively restored CSF drainage capacity in aged mice [24].

**Fig. 2:**
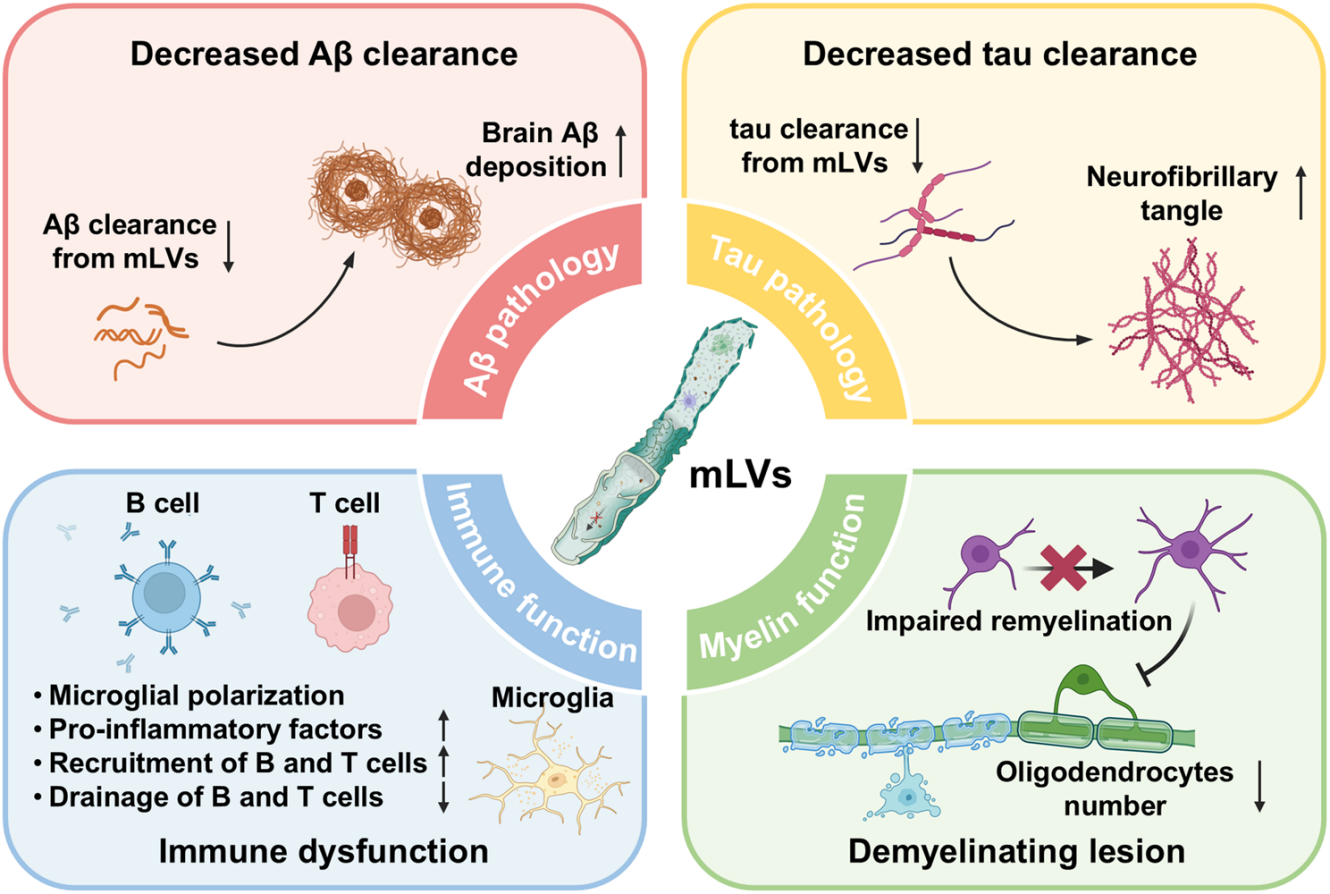
The underlying mechanisms of meningeal lymphatic dysfunction in the pathogenesis of Alzheimer’s disease

Notably, the role of mLVs in Aβ deposition is not straightforward: some studies demonstrated that mLVs ablation exacerbates Aβ pathology [27, 54], while Antila et al. showed that sustained mLVs atrophy or hyperplasia did not significantly alter brain Aβ plaque load in APP/PS1 and 5xFAD mice, despite affecting CSF flow to cervical lymph nodes [28]. To contextualize these mixed findings, a companion commentary by Santisteban and Iadecola [59] noted that compensatory clearance mechanisms—such as blood-brain-barrier transport, enzymatic degradation, or alternative lymphatic routes—may compensate for mLVs dysfunction, and that the contribution of mLVs to AD pathology may be context-dependent, influenced by factors such as disease stage and genetic background. In summary, the lymphatic drainage system of the brain—comprising the GS, mLVs, cervical lymphatic vessels, and CLNs—clears metabolic waste from the brain through a dual mechanism of physical flow and immune degradation, serving as an important pathway for cerebral Aβ clearance (Fig. 2). However, direct clinical evidence of impaired mLVs in AD patients is still lacking, and whether enhancing cervical lymphatic drainage can promote Aβ clearance in the human AD brain remains to be validated. Current research on the causal relationship between mLVs and impaired Aβ clearance is based on animal studies, while human studies remain at the correlative level. Future research should seek clinical evidence from human studies to establish whether meningeal lymphatic dysfunction promotes Aβ deposition.

### Meningeal lymphatic system and tau pathology

Intracellular aggregation of tau protein is another main neuropathological hallmark of AD. It has been proven that misfolded forms of tau can be released into the extracellular space (ISF and CSF), and extracellular tau appears to play an important role in the process of the spread of aggregated tau [60]. Thus, understanding the mechanisms regulating the clearance of extracellular tau from the brain is crucial. Previous research has suggested that intracellular tau is degraded primarily via the autophagy-lysosome and ubiquitin-proteasome systems [61, 62], whereas extracellular tau is primarily cleared by microglial phagocytosis [63, 64]. The discovery of the GS and mLVs has led to the proposal of a novel pathway for extracellular tau clearance [65, 66]. In mice, dysfunction of this integrated glymphatic-mLVs clearance mechanism impairs the removal of extracellular tau from the brain, promoting tau aggregation and the formation of neurofibrillary tangles [65–68]. Within the GS, cerebral arterial pulsation and polarized aquaporin-4 (AQP4) facilitate tau clearance [69–72]. AQP4 facilitates the elimination of extracellular tau from the brain to the CSF and subsequently to deep cervical lymph nodes [66]. Studies in AQP4-knockout mice revealed that AQP4 dysfunction not only impaired glymphatic inflow but also directly or indirectly affected the subsequent mLVs-mediated tau clearance process [66], demonstrating that the GS and mLVs formed a functionally interconnected continuum. Tau retention was significantly longer in mLVs-ablated mice than in wild-type control mice following intracerebral tau injection [73]. Furthermore, the peak plasma tau level appeared later in mLVs-ablated mice than in control mice, indicating that mLVs play a key role in clearing extracellular tau [73]. Long-term cervical lymph node excision (cLE) exacerbated phosphorylated tau accumulation in C57BL/6 mice [67]. A retrospective clinical study revealed an increased risk of dementia in head/neck cancer patients after cervical lymph node dissection, indirectly suggesting that obstruction of cervical lymphatic pathways may be associated with tau pathology [74]. However, whether corresponding alterations occur in mLVs during this process remains unclear. Additionally, in a tauopathy mouse model, increased infiltration of harmful T cells into the brain and enhanced T-cell activation in the meninges were observed [75]. These findings suggest that mLVs may contribute to T-cell infiltration and subsequent neurodegeneration, warranting further investigation. Nevertheless, the present understanding of the integrity of the mLVs in primary tauopathy mice remains limited. Therefore, elucidating the role of mLVs in tau pathology requires assessment of their structural and functional integrity across different models.

Plasma tau levels reflect not only the production and release of tau in the brain but also its clearance efficiency via mLVs [43]. Research has revealed a negative correlation between blood tau levels and meningeal lymphatic function indices, providing human evidence for the association between mLVs and peripheral tau clearance in humans [43]. A study presents the first evidence that p-tau181 is detectable in human CLNs, and that the highest fold difference in corrected concentration between CLNs and blood was observed for p-tau181 [45]. Moreover, the p-tau181 level in CLNs was negatively correlated with age, indicating that there is less lymphatic drainage of brain tau into CLNs with ageing in humans.

The cerebral lymphatic system promotes glymphatic CSF inflow and metabolic clearance during sleep or rest and facilitates CSF drainage via mLVs to CLNs during wakefulness or activity [49, 76]. The sleep‒wake cycle significantly influences tau levels in both mice and humans [77]. Sleep deprivation increases interstitial tau levels by 100% in mice and CSF tau levels by 50% in healthy humans, respectively [77]. However, the regulation of tau protein levels by circadian rhythms remains unclear, and the underlying molecular mechanisms are unclear. Elucidating these mechanisms is essential for understanding how circadian rhythms influence mLVs-mediated tau clearance.

### The meningeal lymphatic system modulates immune function

The immunological mechanisms underlying AD remain incompletely elucidated. The discovery and functional characterization of mLVs have spurred growing research interest in their potential role in AD immunomodulation and disease progression. In animal models, mLVs not only serve as the primary drainage route for immune cells and macromolecules from the brain but also participate in neuroimmune surveillance and the maintenance of brain function [78–82] (Fig. 2). Brain-derived antigens traverse perivascular pathways to access the CSF, where they can be internalized by meningeal and CSF-resident antigen-presenting cells (APCs). Through C-C chemokine receptor type 7 (CCR7)-mediated chemotactic mechanisms, these APCs migrate along mLVs and ultimately accumulate in dCLNs to prime adaptive immune responses [80, 83–85]. Notably, immune cell clusters within the meningeal compartment release soluble cytokines that may penetrate the brain parenchyma via mLVs and the GS, subsequently modulating neuroinflammation, synaptic plasticity, and cognitive function [86–88].

RNA sequencing of LECs from aged mice revealed no significant changes in the expression of classic LEC markers such as Pecam1, Lyve1, Prox1, and Flt4 during ageing; however, alterations were observed in gene sets related to immune and inflammatory pathways [27]. In the meningeal LECs of aged mice, the expression of genes associated with the IFNγ signalling was upregulated, indicating an enhanced response to IFNγ signalling [21]. In the meninges, activated CD4^+^ and CD8^+^ T cells accumulate abnormally and secrete copious amounts of IFNγ, which impairs mLVs integrity through the mislocalization of vascular endothelial-cadherin, a key endothelial adhesion molecule [21]. In AD transgenic mice, Aβ-induced activated astrocytes exhibit a senescence-associated secretory phenotype (SASP) and secrete various inflammatory and inhibitory factors, including thrombospondin-1 (TSP-1) [22]. High expression of TSP-1 inhibits lymphangiogenesis and junctional plasticity via the receptors CD36 and CD47 leading to lymphatic dysfunction [22]. These studies reveal that immune cells and inflammatory responses can directly act on LECs, disrupting the structure and function of mLVs, thereby exacerbating neuroimmune imbalance and contributing to disease progression in both AD and ageing. Notably, in CCR7-deficient AD mice, mLVs morphology remained intact (with normal vessel diameter and coverage), and tracer drainage function was preserved [78]. However, CCR7 deficiency caused immune cell retention, and the resulting abnormal immune milieu could exacerbate cerebral Aβ deposition [78]. These findings indicate that mLVs functionality requires both structural integrity and orchestrated immune cell trafficking.

Impaired mLVs lead to abnormal accumulation of pathological proteins such as Aβ and proinflammatory factors in the brain, thereby broadly activating the innate immune system. After artificial disruption of mLVs, significant Aβ deposition was observed in the meninges of 5xFAD mice, with substantial recruitment and aggregation of macrophages around Aβ plaques [27]. Similarly, damaging mLVs leads to hyperactivation of microglia and astrocytes in the brain, characterized by cell proliferation, morphological changes, and extensive transcriptional reprogramming [54, 89–91]. Upregulated expression of ApoE, Cst7, Spp1, Lpl, and Lyz2 pushes microglia towards a proinflammatory, phagocytically dysfunctional state known as the disease-associated microglia (DAM) phenotype [54]. DAM display impaired phagocytic and antigen-presenting functions alongside sustained inflammatory signalling, which collectively exacerbate neuronal damage and AD progression [54]. Moreover, activated microglia overexpress S100a8/S100a9, which disrupts the excitatory/inhibitory (E/I) synaptic balance by activating both classical and trans-IL-6 signalling pathways, ultimately leading to cognitive decline [89]. Conversely, restoring mLVs structure and function can restore the frequency of miniature inhibitory postsynaptic currents via the microglia-mediated IL-6 pathway, thereby improving learning and memory [89]. Astrocytes also play a significant role in innate immunity. In AD, activated astrocytes not only release IL-1β and IL-6 but also overexpress TSP-1, a key inhibitor of meningeal lymphangiogenesis [22].

mLVs are also involved in AD through the regulation of adaptive immunity. First, studies suggest that detecting the levels of molecules such as Aβ and glial fibrillary acidic protein (GFAP) in dCLNs can reveal whether brain-derived pathological proteins are successfully cleared and delivered to the peripheral immune system, thereby influencing subsequent adaptive immune responses [22]. Second, significantly increased numbers of B cells, CD4⁺ T cells, and CD8⁺ T cells were detected in the meninges and brain parenchyma of aged 5xFAD mice and dCLN-ligated mice [29, 54]. Dysfunctional mLVs impair T-cell drainage, and the accumulated proinflammatory factors and chemokines in the brain recruit peripheral B and T cells into the meninges and parenchyma. This infiltrative process exacerbates mLVs damage, culminating in aggravated neuroinflammation and cognitive decline [92–94]. Notably, T-cell infiltration is markedly elevated in tau pathology-affected brain regions, where these cells undergo a continuum from activation to exhaustion, directly or indirectly inducing neuronal damage through cytotoxic effects and cytokine storm release [75]. Therefore, mLVs dysfunction may promote AD by affecting adaptive immune responses in CLNs and the migration of T and B cells.

In summary, mLVs and dCLNs serve as critical hubs linking the CNS and the peripheral immune system. They play key regulatory roles in essential pathological processes including immune cell migration, immune activation, and neuroinflammation. Impairment of mLVs function disrupts normal immune cell trafficking, leads to aberrant immune activation and allows for neuroinflammation to escalate uncontrollably. Consequently, pathogenic proteins such as Aβ and proinflammatory factors accumulate extensively in the brain, resulting in severe neuronal damage and ultimately accelerating the progression of AD (Fig. 2). The specific molecular mechanisms through which mLVs regulate immune function in AD remain to be fully elucidated, highlighting the need for further research to inform novel therapeutic strategies targeting specific immune cells or inflammatory factors.

### The meningeal lymphatic system regulates myelin function

Myelin, a lipid sheath produced by oligodendrocytes, is essential for normal neuronal function. Both oligodendrocytes and myelination are strongly associated with learning and memory [95–97]. Age-related cognitive decline may arise from the generation of recreated myelin in the brain [98]. In both AD mice and AD patients, myelin structure and function are significantly impaired [99–102]. Single‑cell transcriptomic analyses of AD brain tissue further associate myelination deficits with AD pathophysiology [103, 104]. Aβ deposition contributes to myelin degeneration in AD [100, 101, 105] while myelin dysfunction in turn increases neuronal Aβ production and impairs the clearance of Aβ by microglia, together promoting cerebral Aβ accumulation [102]. Notably, promoting remyelination in AD mice improved cognitive impairment, highlighting myelin repair as a potential therapeutic strategy [105].

Ablation of mLVs in adult mice reduced the number of mature oligodendrocytes and caused myelin dysfunction, likely because the disruption promotes the infiltration of adaptive immune cells into the brain [106]. Furthermore, reduced VEGF‑C levels in the CSF of multiple sclerosis patients suggest impaired immune cell trafficking through mLVs, further supporting the notion that meningeal lymphatic dysfunction may exacerbate cerebral demyelination [106]. These findings collectively indicate a novel role for mLVs in supporting oligodendrocyte survival and maintaining myelination [107]. Consequently, impaired meningeal lymphatic function may promote AD progression by compromising myelin integrity—a mechanism that merits further in‑depth investigation (Fig. 2).

## Targeting the meningeal lymphatic system for AD intervention

### Enhancing mLVs repair through pharmacological modulation

Recent studies suggest that targeting mLVs holds promise as a therapeutic strategy for AD. Given that mLVs development and homeostasis are critically dependent on the VEGF‑C/VEGFR3 signalling pathway [6, 20], modulation of this pathway can effectively promote lymphangiogenesis [108]. Impaired VEGF‑C/VEGFR3 signalling led to mLVs regression and compromised lymphatic drainage to dCLNs [20]. Conversely, VEGF-C supplementation in adult mice induced mLVs formation, increased lymphatic vessel diameter, enhanced cerebral perfusion, and improved macromolecule clearance from CSF [20, 27, 109]. In 5xFAD mice, viral vector‑mediated delivery of VEGF-C not only promoted mLVs growth but also reduced the cerebral Aβ burden, alleviated microglia‑mediated neuroinflammation, and ultimately improved cognitive function [54]. The administration of Yodal, a small‑molecule agonist of Piezo1, reduced intracranial pressure, improved CSF flow, and restored meningeal lymphangiogenesis, dCLN drainage, and brain‑CSF perfusion [110]. Oxytocin treatment effectively restored impaired GS and mLVs structure and function in APP/PS1 mice through multidimensional modulation of cerebral hemodynamics, AQP4 polarity, and meningeal lymphangiogenesis [111]. Atorvastatin ameliorated structural disruption of basal mLVs and lymphatic drainage dysfunction caused by ERK1/2 dephosphorylation in meningeal LECs [112]. Long‑term exercise mitigated AD pathology by modulating astrocyte activation and enhancing lymphatic plasticity and drainage function [22]. Traditional Chinese medicine provides unique molecular resources for lymphatic regulation and dementia treatment [108, 113–115]. Borneol increases mLVs permeability and lumen diameter, thereby enhancing meningeal lymphatic drainage, promoting cerebral Aβ clearance, and reducing pathological changes and cognitive deficits [116, 117]. Additionally, compound formulas such as Xueshuantong ameliorate lymphatic structural defects and facilitate Aβ clearance in AD mice [20]. Beyond promoting neovascularization, improving the drainage efficiency of existing lymphatic vessels represents another effective approach. Topical application of prostaglandin F2α (PGF2α) to cervical lymphatic vessels in aged mice promoted calcium influx in smooth muscle cells, increasing the frequency of lymphatic contraction by 152.0% and accelerating the velocity of lymph flow by 3.5‑fold [24].

### Enhancing mLVs repair through physical approaches

Physical methods, including light, magnetic, and mechanical stimulation, can regulate both the microenvironment and functional activity of the lymphatic system. Photobiomodulation (PBM), a noninvasive intervention utilizing visible and near‑infrared light at non‑thermal, low‑level doses to regulate mitochondrial function, has demonstrated neuroprotective potential. Recent research suggests that the enhancement of meningeal lymphatic drainage may constitute a pivotal therapeutic mechanism underlying PBM [118], offering a novel scientific perspective for intervening in AD. In AD animal models, eight‑day transcranial PBM treatment increased the lymphatic clearance of CSF solutes by up to 9‑fold and significantly reduced Aβ deposition in the cortex and hippocampus [119]. Notably, the timing of light intervention is important: compared with daytime intervention, nighttime transcranial light stimulation was more effective at promoting dural lymphatic flow to dCLNs, enhancing Aβ clearance, and improving cognitive function [120]. Studies on light at specific frequencies, particularly 40 Hz, have revealed multiple mechanisms underlying its ability to promote lymphatic clearance [121]. Murdock et al. demonstrated that combined 40 Hz visual and auditory stimulation effectively promoted Aβ clearance via mLVs in 5xFAD mice [53]. This effect may involve the activation of vasoactive intestinal peptide‑expressing interneurons and the subsequent modulation of arterial pulsation [53]. Notably, when lymphatic clearance was inhibited, the Aβ‑clearing effect of this multisensory stimulation was abolished, confirming its dependence on a functional lymphatic system [53]. Furthermore, 40 Hz flickering light stimulated adenosine signalling, enhanced cerebrovascular pulsatility, and promoted AQP4 polarization on astrocytic end‑feet, thereby driving lymphatic flow to augment waste clearance [122].

Different wavelengths and modalities of light‑based strategies collectively expand the scope of phototherapy. Research has shown that two‑week 40 Hz blue light therapy activated the specific vLGN/IGL‑Re neural circuit and modulated AQP4 polarity, directly guiding Aβ drainage via the mLVs to dCLNs and consequently reducing brain Aβ deposition and cognitive impairment in 5xFAD mice [123]. In terms of clinical relevance, combining this 40 Hz blue light with low‑dose anti‑Aβ antibody synergistically alleviated Aβ deposition and improved cognition, providing key evidence for the combined use of phototherapy and biological agents [123]. Another study employed 808 nm near‑infrared transcranial therapy in aged and AD mice, and revealed that improving mitochondrial metabolism in LECs reduced brain Aβ deposition, neuroinflammation, and other AD‑related pathologies, thereby ameliorating cognitive deficits [124]. In summary, phototherapy across different wavelengths and frequencies can enhance meningeal lymphatic drainage through direct modulation of the GS/mLVs, ultimately facilitating Aβ clearance and cognitive improvement.

Cranial bone manoeuvres could improve meningeal lymphatic drainage function through upregulating VEGF‑C/VEGFR3 expression and subsequently reducing brain Aβ deposition and alleviating neuroinflammation [125]. Percutaneous mechanical manipulation of superficial cervical lymphatic vessels using force‑control devices doubled CSF outflow and corrected CSF drainage dysfunction in aged mice [126]. Repetitive transcranial magnetic stimulation (rTMS) has also been shown to increase the drainage efficiency of mLVs and attenuate AD‑related pathology in mice [127]. The therapeutic strategy of focused ultrasound (FUS) combined with microbubbles to open the blood‑brain barrier can alter the kinetics of solute exchange between brain tissue and CSF, increase Aβ drainage into the CSF, and ultimately accelerate its clearance via lymphatic pathways [128]. As a traditional physical stimulation modality, eight weeks of electroacupuncture improved lymphatic clearance and AQP4 polarization on astrocytic end-feet in SAMP8 mice, leading to reduced Aβ deposition and enhanced cognitive function [129].

**Fig. 3:**
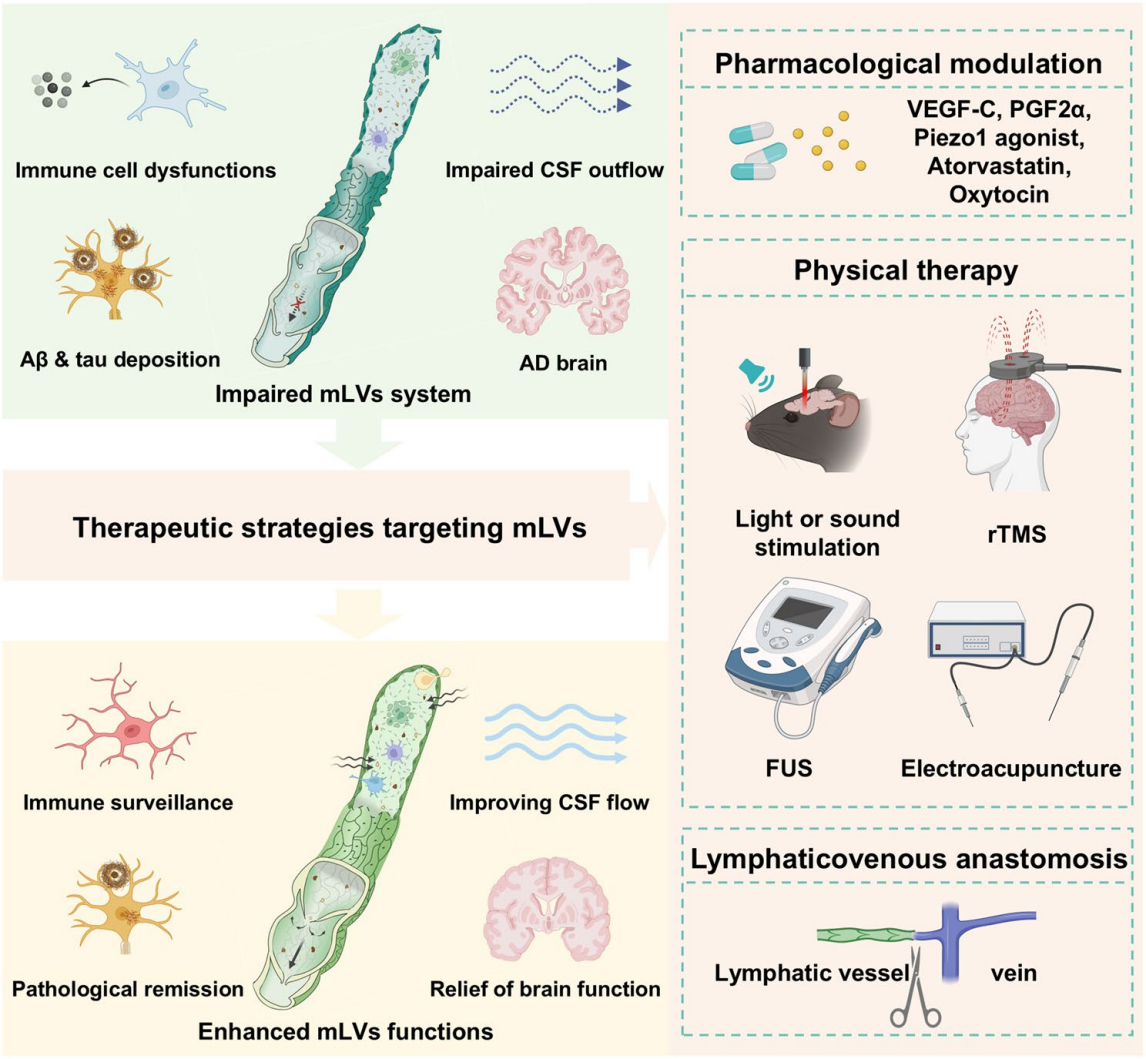
Intervention for Alzheimer’s disease by targeting meningeal lymphatic vessels. AD: Alzheimer’s disease; rTMS: repetitive transcranial magnetic stimulation; FUS: focused ultrasound

In conclusion, potential strategies for modulating mLVs in AD intervention include directly targeting lymphangiogenesis and lymphatic contraction via chemical approaches or indirectly improving the intracranial environment and drainage dynamics through physical modalities (Fig. 3). Future research should focus on optimizing light‑based parameters and conducting clinical studies to validate their efficacy in AD.

### Lymphaticovenous anastomosis

Lymphaticovenous anastomosis (LVA) is a microsurgical procedure for treating lymphedema [130]. Its mechanism involves establishing anastomoses between superficial lymphatic vessels and adjacent venules using supermicrosurgical techniques, thereby shunting lymphatic fluid into the venous circulation to enhance lymphatic return and mitigate lymphedema [131, 132]. Deep cervical lymphaticovenous anastomosis (dcLVA) represents an emerging surgical approach developed in recent years that involves anastomosing bilateral cervical lymphatic vessels with veins to facilitate meningeal lymphatic drainage into the venous system, with the goal of improving metabolic waste clearance in the brain [133, 134]. In 2020, Chinese researchers performed this procedure on an elderly patient with cognitive impairment. Postoperatively, the patient exhibited reduced lymphatic reflux pressure and improved cognitive function [135]. Currently, multiple hospitals in China are conducting clinical trials to evaluate the safety and efficacy of dcLVA for moderate-to-severe AD (Table 1). Notably, although China pioneered the AD lymphatic drainage concept, its clinical translation has been temporarily halted because of increasingly stringent domestic regulatory requirements. Conversely, Singaporean researchers registered a clinical trial for dcLVA on January 17, 2025, followed by their American counterparts on September 9, 2025 (Table 1).

**Table 1:** Clinical trials evaluating the safety and efficacy of dcLVA for AD intervention are being conducted

Item	Study Type	Study Site	Study Start	Enrollment	Eligibility Criteria	Interventions (Intervention Group)	Interventions (Control Group)	Primary Outcomes
ChiCTR2400094603	Intervention	China	2024/8/1	100	Moderate to severe AD	dcLVA	Non-surgical treatment	MMSE, ADAS Cog, ADL
ChiCTR2400089883	Intervention	China	2024/10/1	30	Mild to moderate AD	dcLVA	None	ADL
ChiCTR2500095309	Intervention	China	2025/1/8	100	Moderate to severe AD	dcLVA	None	CDR, MMSE, MoCA, ADL
ChiCTR2500098356	Intervention	China	2025/3/1	50	All degrees of AD	dcLVA	None	PET/CT
ChiCTR2500097585	Intervention	China	2025/3/1	60	Moderate to severe AD	dcLVA	Non-surgical treatment	MMSE, MoCA, ADL, Aβ, tau
ChiCTR2500104509	Intervention	China	2025/4/26	10	Moderate to severe AD	dcLVA	None	CFA, ADL, MRI
ChiCTR2500101614	Intervention	China	2025/4/30	10	Moderate to severe AD	dcLVA	None	HKBC, ADL
ChiCTR2500102667	Intervention	China	2025/5/30	92	Moderate to severe AD	dcLVA + Donepezil 10 mg/d	Donepezil 10 mg/d	CFA
ChiCTR2500105493	Intervention	China	2025/6/1	20	All degrees of AD	dcLVA	Non-surgical treatment	Blood oxygen signal, MMSE, ADL, ADAS Cog
ChiCTR2400084617	Intervention	China	2023/12/1	10	All degrees of AD	dcLVA	Non-surgical treatment	Neuropsychological Scale Test, Neuroimaging examination, Cerebrospinal fluid examination
ChiCTR2500105876	Observation	China	2025/2/15	20	All degrees of AD with dcLVA	NA	NA	MMSE
ChiCTR2500099855	Intervention	China	2025/3/1	30	Moderate to severe AD	dcLVA	Non-surgical treatment	CDR-SB, Blood biomarkers (Aβ42/p-tau181, p-tau 181, p-tau217), SAEs
ChiCTR2500102675	Intervention	China	2025/4/1	70	Mild to moderate AD	dcLVA	Traditional medication	CDR-SB, PET/CT, ADL, Brain atrophy rate
ChiCTR2500106464	Observation	China	2025/7/1	20	Moderate to severe AD with dcLVA	NA	NA	Neuropsychological assessment
NCT06706947	Intervention	China	2024/12/1	20	Mild to moderate AD	dcLVA	None	MMSE
NCT07094438	Intervention	China	2025/1/1	98	Moderate AD	dcLVA	None	MMSE, CDR, ADAS Cog
NCT06965062	Intervention	Singapore	2025/4/1	10	Mild to moderate AD	dcLVA	None	Treatment-related adverse events, MMSE, MoCA, CDR, NPI, GDS-SF, QoL-AD, ZBI, BADL, BGA
NCT06918145	Intervention	China	2025/4/2	80	All degrees of AD	dcLVA	None	MMSE
NCT06978946	Intervention	China	2025/5/28	85	Moderate to severe AD	dcLVA	None	CDR-SB
NCT07178210	Intervention	America	2025/12/1	15	Mild to moderate AD	Use of symani surgical system for performing microsurgical techniques in dCLNs	None	Device-related serious adverse events at 30-days
NCT06530732	Intervention	China	2024/7/1	60	All degrees of AD	dcLVA + Lecanemab	Lecanemab	CDR
NCT06448442	Observation	China	2024/8/1	8	Mild to moderate AD with dcLVA	NA	NA	ADAS Cog
NCT06936514	Intervention	China	2025/1/8	45	Moderate to severe AD	dcLVA	Non-surgical treatment	ADAS-Cog, MoCA, MMSE, CDR-SB, CDR-GS
NCT06852352	Intervention	China	2025/2/28	160	MCI	dcLVA	None	CDR-SB, MMSE, MoCA, ADL
NCT07058129	Observation	China	2025/7/30	814	All degrees of AD with dcLVA	NA	NA	CDR-SB, Incidence of perioperative complications
NCT07060391	Intervention	China	2025/7/31	186	Mild to moderate AD	dcLVA	Donepezil drug treatment	ADAS Cog
NCT07073066	Intervention	China	2025/11/30	376	Moderate to severe AD	dcLVA + Usual care	Usual care	CDR-SB
NCT07294885	Intervention	China	2026/3/20	50	Mild to moderate AD	dcLVA	Sham surgery	MMSE, MoCA, BI, CIBIC-plus
NCT07458620	Intervention	China taiwan	2026/3	35	All degrees of AD	dcLVA	None	SAEs

The data were from https://www.chictr.org.cn and https://clinicaltrials.gov. ADAS Cog: Alzheimer’s Disease Assessment Scale Cognitive Scale; ADL: Activities of Daily Living Scale; BADL: Basic and Instrumental Activities of Daily Living; BGA: Basic Gait Assessment; BI: Changes in Barthel index; CDR-SB: Clinical Dementia Rating Scale - Sum of Boxes; CDR-GS: Change in Clinical Dementia Rating Scale global score; CFA: Cognitive Function Assessment; dCLNs: Deep cervical lymph nodes; CIBIC-plus: Changes in Clinician’s Interview-Based Impression of Change-plus caregiver input; dcLVA: Deep cervical lymphaticovenous anastomosis; GDS-SF: Geriatric Depression Scale-Short Form; HKBC: Hong Kong Brief Cognitive Test; MMSE: Mini-Mental State Examination; MoCA: Montreal Cognitive Assessment; NPI: Neuropsychiatric Inventory; PET/CT: Positron Emission Tomography/Computed Tomography; QoL-AD: Quality of Life-Alzheimer’s Disease; SAEs: Incidence of serious adverse events; ZBI: Zarit Burden score

However, the theoretical and experimental basis for the application of dcLVA in AD treatment remains insufficient. The intended goal of dcLVA is to accelerate meningeal and cervical lymphatic drainage thereby promoting the clearance of AD pathogenic proteins from the brain. Nonetheless, the contribution of mLVs to AD pathology and cognitive decline cannot be oversimplified [27, 28, 54]. Other critical unanswered questions include whether cervical lymphatic drainage is impaired in AD patients, whether meningeal lymphatic pressure exceeds venous pressure, and whether dcLVA truly enhances meningeal lymphatic function. Current clinical trial findings remain conflicting regarding whether dcLVA can mitigate cerebral Aβ deposition and ameliorate cognitive function in patients with AD. A study of 26 AD patients found patients’ that Mini-Mental State Examination (MMSE) scores rose significantly one month after dcLVA, whereas Montreal Cognitive Assessment (MoCA) scores and CSF biomarkers (Aβ_42_, Aβ_42_/Aβ_40_ ratio, p-tau, t-tau) showed no notable changes [136]. A recent study observed decreased CSF Aβ42, Aβ40, and p-tau levels postoperatively in severe AD patients, along with increased plasma concentrations of these AD biomarkers and modest cognitive improvements over a 6-month follow-up period [137]. A case report details that a female AD patient who underwent dcLVA saw improved MMSE and MoCA scores postoperatively, with PET/CT imaging showing reduced brain Aβ deposition four months after surgery [138]. However, all existing clinical studies on dcLVA in AD are limited by single-arm, non-randomized designs, lack of blinding, small sample sizes, and short follow-up durations, precluding definitive conclusions about efficacy or causality. Well-powered, randomized controlled trials with long-term follow-up and objective outcome measures are urgently needed to validate the therapeutic potential of dcLVA in AD. Therefore, future research should further investigate the effects of LVA on AD pathology and cognitive function through additional preclinical and clinical studies while also establishing more robust institutional frameworks to support the translation of such innovative therapeutic concepts.

## Conclusions and perspectives

This review systematically summarizes the potential role of mLVs in AD and highlights potential therapeutic strategies targeting this pathway. As a putative principal route for metabolic waste clearance and neuroimmune modulation in the brain, mLVs dysfunction has been reported to exacerbates Aβ and tau deposition, disrupts neuroimmune homeostasis, and impairs myelin integrity—collectively contributing to AD pathogenesis in preclinical models. Emerging interventions—such as VEGF‑C‑mediated lymphangiogenesis promotion, photobiomodulation and magnetic stimulation to enhance lymphatic drainage, and surgical dcLVA—collectively underscore the therapeutic potential of targeting mLVs in AD.

The discovery of mLVs has challenged long-standing neurobiological paradigms. Recent preclinical studies have validated the involvement of mLVs in AD-related pathological cascades, increasing our mechanistic understanding of disease pathogenesis and accelerating the development of novel AD interventions. Nevertheless, critical knowledge gaps persist in this rapidly evolving field. First, although existing evidence indicates that mLVs participate in Aβ/tau clearance and neuroimmune modulation, the precise functional significance and mechanistic underpinnings of these processes remain incompletely defined. Second, the molecular mechanisms and systemic regulators of mLVs dysfunction are not fully understood; future studies should investigate how comorbid systemic diseases impact the structure and function of mLVs. Third, most research on meningeal lymphatic dysfunction in AD derives from preclinical models, and to date, no study has directly assessed mLVs function in AD patients. It is therefore important to interpret the current evidence with caution. Prospective cohort studies are needed to directly evaluate mLVs function in AD patients and to examine its correlations with AD biomarkers and clinical disease progression. Fourth, well-powered, rigorously controlled clinical trials are necessary to determine whether enhancing meningeal lymphatic function affects AD pathology or preserves cognitive function. Fifth, the development of reliable imaging and fluid biomarkers for precise assessment of mLVs and cervical lymphatic vessel function is needed.

## Data Availability

No datasets were generated or analysed during the current study.

## Abbreviations

AD: Alzheimer’s disease
ADAS Cog: Alzheimer’s disease assessment scale cognitive scale
ADL: Activities of daily living scale
APCs: Antigen-presenting cells
AQP4: Aquaporin-4
Aβ: Amyloid-β
BADL: Basic and instrumental activities of daily living
BGA: Basic Gait Assessment
CCR7: C-C chemokine receptor type 7
CDR-SB: Clinical Dementia Rating Scale-Sum of Boxes
CFA: Cognitive Function Assessment
Cle: Cervical lymph node excision
CLNs: Cervical lymph nodes
CNS: Central nervous system
CSF: Cerebrospinal fluid
DAM: Disease-associated microglia
DCE-MRI: Dynamic contrast-enhanced magnetic resonance imaging
dCLNs: Deep cervical lymph nodes
dcLVA: Deep cervical lymphaticovenous anastomosis
DSCR1: Down syndrome critical region 1
DTI-ALPS: Diffusion tensor imaging analysis along the perivascular space
FLAIR: Fluid-attenuated inversion recovery
FUS: Focused ultrasound
GDS-SF: Geriatric Depression Scale-Short Form
GFAP: Glial fibrillary acidic protein
GS: Glymphatic system
HKBC: Hong Kong Brief Cognitive Test
LECs: Lymphatic endothelial cells
LVA: Lymphovenous anastomosis
mLVs: Meningeal lymphatic vessels
MMSE: Mini-Mental State Examination
MoCA: Montreal Cognitive Assessment
MRI: Magnetic resonance imaging
NPI: Neuropsychiatric Inventory
PBM: Photobiomodulation
PD: Parkinson’s disease
PET/CT: Positron Emission Tomography/Computed Tomography
PGF2α: Prostaglandin F2α
QoL-AD: Quality of Life-Alzheimer’s Disease
rhVEGF-C: Recombinant human vascular endothelial growth factor C
rTMS: Repetitive transcranial magnetic stimulation
SASP: Senescence-associated secretory phenotype
sICH: Spontaneous intracerebral hemorrhage
T1WI BB MRI: T1-weighted black-blood magnetic resonance imaging
TSP-1: Thrombospondin-1
VEGF-C: Vascular endothelial growth factor C
VEGFR-3: Vascular endothelial growth factor receptor 3
ZBI: Zarit Burden score
